# Determinants of adherence to daily PrEP measured as intracellular tenofovir diphosphate concentrations over 24 months of follow-up among men who have sex with men

**DOI:** 10.1136/sextrans-2022-055499

**Published:** 2022-09-05

**Authors:** Mark AM van den Elshout, Elske Hoornenborg, Liza Coyer, Peter L Anderson, Udi Davidovich, Henry JC de Vries, Maria Prins, Maarten F Schim van der Loeff

**Affiliations:** 1 Department of Infectious Diseases Research, Public Health Service Amsterdam, Amsterdam, The Netherlands; 2 Department of Pharmaceutical Sciences, University of Colorado—Anschutz Medical Campus, Aurora, Colorado, USA; 3 Department of Social Psychology, University of Amsterdam Faculty of Social and Behavioural Sciences, Amsterdam, The Netherlands; 4 Amsterdam Institute for Infection & Immunity (AII), Amsterdam UMC Locatie AMC, Amsterdam, The Netherlands

**Keywords:** treatment adherence and compliance, HIV, pre-exposure prophylaxis, cohort studies

## Abstract

**Objectives:**

Adherence is key to the effectiveness of oral pre-exposure prophylaxis (PrEP) to prevent HIV. Therefore, we aimed to explore factors associated with adherence to daily PrEP (dPrEP).

**Methods:**

Men who have sex with men (MSM) using dPrEP (emtricitabine/tenofovir disoproxil) within the Amsterdam PrEP demonstration project at the Public Health Service of Amsterdam, provided dried blood spots (DBS) 12 and 24 months after PrEP initiation. From DBS, we determined intracellular tenofovir diphosphate (TFV-DP) concentrations to assess adherence; TFV-DP ≥700 fmol/punch was considered adequate. We assessed associations of sociodemographic, clinical and behavioural characteristics with TFV-DP concentrations using multivariable linear regression.

**Results:**

Of 263 participants who attended 12-month or 24-month study visits while on dPrEP, 257 (97.7%) provided DBS at one or both visits (492 DBS in total). Median TFV-DP concentration was 1299 (IQR 1021–1627) fmol/punch (12 months: 1332 (1087–1687); 24 months: 1248 (929–1590]). Higher TFV-DP concentrations were associated with: older age (p=0.0008), condomless anal sex with a casual partner in 6 months preceding PrEP initiation (+166 fmol/punch; 95% CI 36.5 to 296) and using a mobile application providing visualised feedback on PrEP use and sexual behaviour (+146 fmol/punch; 95% CI 28.1 to 263). Lower TFV-DP concentrations were associated with longer duration of PrEP use (24 vs 12 months; −91.5 fmol/punch; 95% CI −155 to −28.1). Time-updated number of sex partners, diagnosed STIs and chemsex were not associated with TFV-DP concentrations.

**Conclusions:**

Overall, TFV-DP concentrations were high among MSM using dPrEP, indicating excellent adherence. Especially older participants, those who reported condomless anal sex with a casual partner prior to PrEP initiation and those who used an app with visualised feedback showed higher levels of adherence. As TFV-DP concentrations had decreased slightly at 2 years of PrEP use when compared with 1 year, we emphasise the importance of adherence counselling to those who continue using PrEP.

**Trial registration number:**

NL5413.

**WHAT IS ALREADY KNOWN ON THIS TOPIC**

- Dried blood spots can be used to assess adherence objectively via intracellular drug levels of tenofovir diphosphate (TFV-DP), which have long half-lives.

**WHAT THIS STUDY ADDS**

- TFV-DP concentrations were especially high among older participants, those reporting condomless anal sex before initiating pre-exposure prophylaxis (PrEP) and those using a mobile application which provides visualised feedback on sexual behaviour and PrEP use.
- Adherence slightly declined between 12 and 24 months.

**HOW THIS STUDY MIGHT AFFECT RESEARCH, PRACTICE OR POLICY**

- A mobile application could help improve adherence.
- Adherence slightly declines over time, which emphasises the importance of continued attention to adherence over the course of people’s PrEP careers.

## Introduction

Pre-exposure prophylaxis (PrEP) and other HIV prevention strategies are part of an effort to eliminate new HIV infections, since a cure is not available and HIV continues to affect the health of millions of people globally. In the Netherlands, the number of new HIV diagnoses has been decreasing in the past decade. In 2010, almost a 1000 HIV infections were newly diagnosed in the Netherlands; in 2020, this was reduced to 411. Men who have sex with men (MSM) remain disproportionally affected, accounting for 63% of newly diagnosed infections in 2020.^1^

Adequate adherence to PrEP is key to its effectiveness in preventing HIV.^2^ Therefore, WHO advises brief adherence counselling at each PrEP visit.^3^ Insight into determinants of PrEP adherence may help clinicians to focus their counselling efforts on those who are more likely to be less adherent, thus improving adherence outcomes and reducing HIV transmission.

Adherence can be measured in various ways. Self-reported adherence may be unreliable due to recall and social-desirability bias. Plasma drug levels have the disadvantage of a short half-life, so a single measure cannot be used to assess adherence over longer time periods, which is crucial for PrEP effectiveness. Another way is measuring the intracellular tenofovir diphosphate (TFV-DP) concentrations in red blood cells. Using this intracellular metabolite to measure averaged PrEP adherence has the benefit of a ~25-fold longer half-life compared with plasma drug levels. Thus, gradients of adherence can be ascertained over the preceding 4–6 weeks instead of yes-or-no dosing over the last few days, analogous to haemoglobin A1C for glucose.^4 5^

The Amsterdam PrEP demonstration project (AMPrEP) aimed to assess the acceptability and feasibility of PrEP for MSM and transgender people in Amsterdam, the Netherlands. At that time, PrEP was not yet available through regular healthcare. In AMPrEP, we collected dried blood spots (DBS) to measure TFV-DP concentrations at months 12 and 24 after PrEP initiation, and detailed behavioural information at each 3-monthly study visit.

The aim of the present study is to assess determinants of long-term adherence to daily PrEP (dPrEP), by examining the associations between demographic, clinical and behavioural characteristics and TFV-DP concentrations among participants of the AMPrEP cohort over the first 2 years of follow-up.

## Methods

### Settings and location of data collection

AMPrEP (3 August 2015–1 December 2020) was an open-label demonstration study including MSM and transgender women (TGW) who were offered a free-of-charge coformulation of emtricitabine and tenofovir disoproxil 200/245 mg, to be used as dPrEP or event-driven PrEP (edPrEP). Study design, aim and procedures have been described previously.^6^ Briefly, participants attended 3-monthly study visits at the Centre of Sexual Health of the Public Health Service of Amsterdam, the Netherlands. Eligible were HIV-negative MSM and TGW, who were ≥18 years old and in the 6 months prior to AMPrEP screening had a substantial likelihood to acquire HIV sexually.^6^ Switching between dPrEP and edPrEP was allowed at each 3-monthly study visit. If participants had switched regimens in-between study visits, the switch would be registered at the next study visit. All AMPrEP participants provided blood samples for HIV and STI testing at each study visit and for making DBS at the 12 and 24 months study visit. A subgroup of participants was included in a nested randomised clinical trial (RCT) assessing the effect of an app providing visualised feedback on good adherence (TFV-DP ≥700 fmol/punch), as reported previously.^7^ For inclusion into the RCT participants had to use dPrEP at the moment of inclusion (between 4 May 2016 and 12 September 2016) and be willing to use the app (RCT protocol available online via https://www.trialregister.nl/trial/5413). For the present analyses, we only used data from AMPrEP participants using dPrEP at the moment of DBS sampling.

### Measures

Sociodemographic, psychosocial, clinical and behavioural characteristics were collected via questionnaires. Demographics collected at inclusion in AMPrEP were age, gender identity, ethnicity, place of residency, education level, employment status, income level, living situation, relationship status and sexual preference. Behavioural and clinical characteristics included history of condomless anal sex and bacterial STIs in the 6 months prior to inclusion. Self-reported number of sex partners and sex episodes, including partner type and condom use, were recorded 3 monthly. For psychosocial determinants, sexual compulsivity was measured using the sexual compulsivity scale,^8^ with a score ≥24 being indicative of a greater impact of sexual thoughts on daily functioning and of an inability to control sexual thoughts or behaviours.^9^ Chemsex was defined as the use of γ-hydroxybutyrate/γ-butyrolactone, methamphetamine or mephedrone prior to or during sex. Symptoms of depression or anxiety were assessed using the Mental Health Inventory-5 (MHI-5) score, where a score of <60 indicated symptoms of depression or anxiety.^10 11^ The Alcohol Use Disorders Identification Test (AUDIT)^12^ and Drug Use Disorder Identification Test (DUDIT)^13^ questionnaires were used to assess problematic alcohol and drug use, respectively; scores ≥8 are interpreted as indicative of alcohol-related or drug-related problems.^14^ We assessed sexual compulsivity, chemsex, MHI-5, AUDIT and DUDIT yearly. Level of concern about acquiring HIV and level of importance to prevent HIV were collected on 7-point Likert scale. Level of concern was dichotomised post hoc to low (scores 1–2) vs neutral to high (scores 3–7) and importance to prevent HIV was dichotomised to very unimportant to important (scores 1–6) vs very important (score 7), both due to skewed data.^7^

### Laboratory methods

Whole blood collected via phlebotomy by a research nurse or physician was spotted onto Whatman 903 Protein Saver Cards (GE Healthcare Bio-Sciences, Piscataway, New Jersey, USA) by a lab technician. After drying for at least 2 hours at room temperature, the cards were sealed in plastic bags including a desiccant with a humidity indicator (MiniPax, Multisorp Technologies, Buffalo, New York, USA) and stored at −20°C. Samples were shipped on dry ice in batches for analysis at the Colorado Antiviral Pharmacology Laboratory (University of Colorado Anschutz Medical Campus, Aurora, Colorado, USA), as described previously.^5 15^ Intracellular TFV-DP concentrations were reported as fmol/3 mm punch. Results were not provided to participants.

### Statistical methods

Study visits at which participants provided DBS samples were included if participants had used dPrEP in the prior 3 months and had not used direct-acting antivirals (DAAs) for treatment of hepatitis C virus infection between enrolment and the 24-month visit. The latter condition was necessary, because combined use of sofosbuvir containing DAA regimens and tenofovir is associated with elevated intracellular TFV-DP concentrations.^16^ A multivariable linear regression model using generalised estimating equations assessed the association between baseline factors and characteristics during follow-up (sexual behaviour, mental health and chemsex), and the outcome TFV-DP concentrations. Variables with βs in univariable analysis with p<0.25 were initially included, subjected to backward selection and kept in the multivariable model if p<0.05. Access to either the basic app or the app providing feedback was included in the multivariable model a priori.

We reran the above-mentioned model in a sensitivity analysis by excluding edPrEP users (already excluded from primary analyses), and participants who had been using dPrEP until the sampling date, but switched to the event-driven regimen on the DBS sampling visit; hereby variables from the model obtained in the main analysis were included a priori and other variables were selected using forward selection and included if p<0.05. This sensitivity analysis was done because clinical experience taught us that participants–for whom a switch to edPrEP was recorded at a visit—had often already switched to edPrEP in the previous weeks/months; in these participants lower TFV-DP concentrations are expected, which does not necessarily indicate poor adherence.

Statistical analyses were performed in STATA V.15.1 (StataCorp, College Station, Texas, USA).

The study was registered at the Netherlands Trial Register (NL5413).

### Role of funding source

The funders played no role in the study design, data collection, data analysis, data interpretation or writing of the manuscript. The authors had full access to all data and were responsible for the decision to submit the manuscript for publication.

## Results

### Participants

Of 367 AMPrEP participants with any follow-up, 20 (5.4%) did not attend the 12-month or 24-month study visit. Of 347 that did, 74 (21.3%) were using edPrEP prior to both sampling visits, 6 (1.7%) did not provide DBS and 7 (2.0%) had been using DAAs in the study period (online supplemental figure). During follow-up, two participants withdrew informed consent and were excluded from all analyses. One participant was considered an outlier, because in both DBS the TFV-DP concentration levels exceeded 4000 fmol/punch, and was also excluded. Finally, 257 participants with a DBS sample at one or both visits (492 DBS in total) while on dPrEP were included in the analysis. The study visits with DBS samples took place between 28 July 2016 and 19 July 2018. No HIV infections were diagnosed in the study population in this time period.

10.1136/sextrans-2022-055499.supp1 Supplementary data

### Baseline characteristics

Median age was 39 years (IQR 32–48; table 1). One participant identified as TGW, 256 as male. Ethnicity was self-declared as white by 84%. Three-quarters of participants reported their education level as college/university. Sexual preference was described as exclusively homosexual by 78% of participants. In the 3 months prior to inclusion in AMPrEP, the median number of sex partners was 15 (IQR 8–32) and the median number of condomless anal sex episodes with casual partners 6 (IQR 3–15). In the 6 months prior to inclusion, 39% of participants were diagnosed with a bacterial STI and 95% reported condomless anal sex with a casual partner (table 1).

**Table 1:** Behavioural and psychosocial characteristics and baseline demographic variables of daily PrEP users

	Total (n=257)	
	N	%*
Demographic characteristics		
Age (years)		
Median (IQR)	39	(32–48)
Age (years; categorised)		
≤34	85	33
35–44	80	31
≥45	92	36
Gender identity		
Male	256	99
Transgender woman	1	1
Self-declared ethnicity		
White	217	84
Non-white	40	16
Place of residency in the Netherlands		
Amsterdam	159	62
Other	98	38
Education level		
No college/University	64	25
College/University	193	75
Employment†		
Employed	202	80
Unemployed	9	4
Other (retired, volunteer, disabled, student)	43	17
Net monthly income in €‡		
≤1700	66	27
1701–2950	103	42
>2950	74	30
Living situation		
Alone	141	55
With partner	81	32
With parents/Flatmates	35	14
Steady relationship§		
No	143	56
Yes	111	44
Sexual preference¶		
Exclusively homosexual	199	78
Not exclusively homosexual	57	22
Sexual behaviour		
Number of sex partners**		
Median (IQR)	15	(8–32)
Number of condomless anal sex episodes with casual partners**		
Median (IQR)	6	(3–15)
STIs††		
No	158	61
Yes	99	39
Condomless anal sex with a casual partner (6 months prior to inclusion in AMPrEP)		
No	13	5
Yes	244	95
Mental health characteristics and drug use		
Sexual compulsivity scale		
Score <24 (no indication of sexual compulsivity)	196	76
Score ≥24 (indication of sexual compulsivity)	61	24
Chemsex‡‡		
No	147	58
Yes	106	42
Depression or anxiety symptoms		
MHI-5 score ≥60 (no symptoms)	212	82
MHI-5 score <60 (symptoms)	45	18
Alcohol Use Disorder Identification Test§		
Score <8 (no indication)	186	73
Score ≥8 (indication)	68	27
Drug Use Disorder Identification Test		
Score <8 (no indication)	160	62
Score ≥8 (indication)	97	38
Level of concern about acquiring HIV§§		
Low	41	16
Neutral to high	216	84
Level of importance to prevent HIV§§		
Very unimportant to important	70	27
Very important	187	73
Access to mobile application		
Standard app	152	59
Extended app	105	41
AMPrEP study visit attendance		
12 months	246	96
24 months	215	84

AMPrEP study, Amsterdam, 2015–2018.

*Percentages may not total 100 due to rounding.

†3 missing.

‡14 missing.

§3 missing.

¶1 missing.

**In the 3 months prior to inclusion into AMPrEP.

††Chlamydia, gonorrhoea and/or syphilis within 6 months prior to inclusion into AMPrEP.

‡‡Use of γ-hydroxybutyrate, γ-butyrolactone, methamphetamine or mephedrone prior to or during sex in the 3 months prior to inclusion into AMPrEP, 4 missing.

§§Scale 1–7, dichotomised.

AMPrEP, Amsterdam PrEP demonstration project; MHI5, Mental Health Inventory-5; PrEP, pre-exposure prophylaxis; RCT, randomised clinical trial.

### Intracellular TFV-DP concentrations

**Figure 1:**
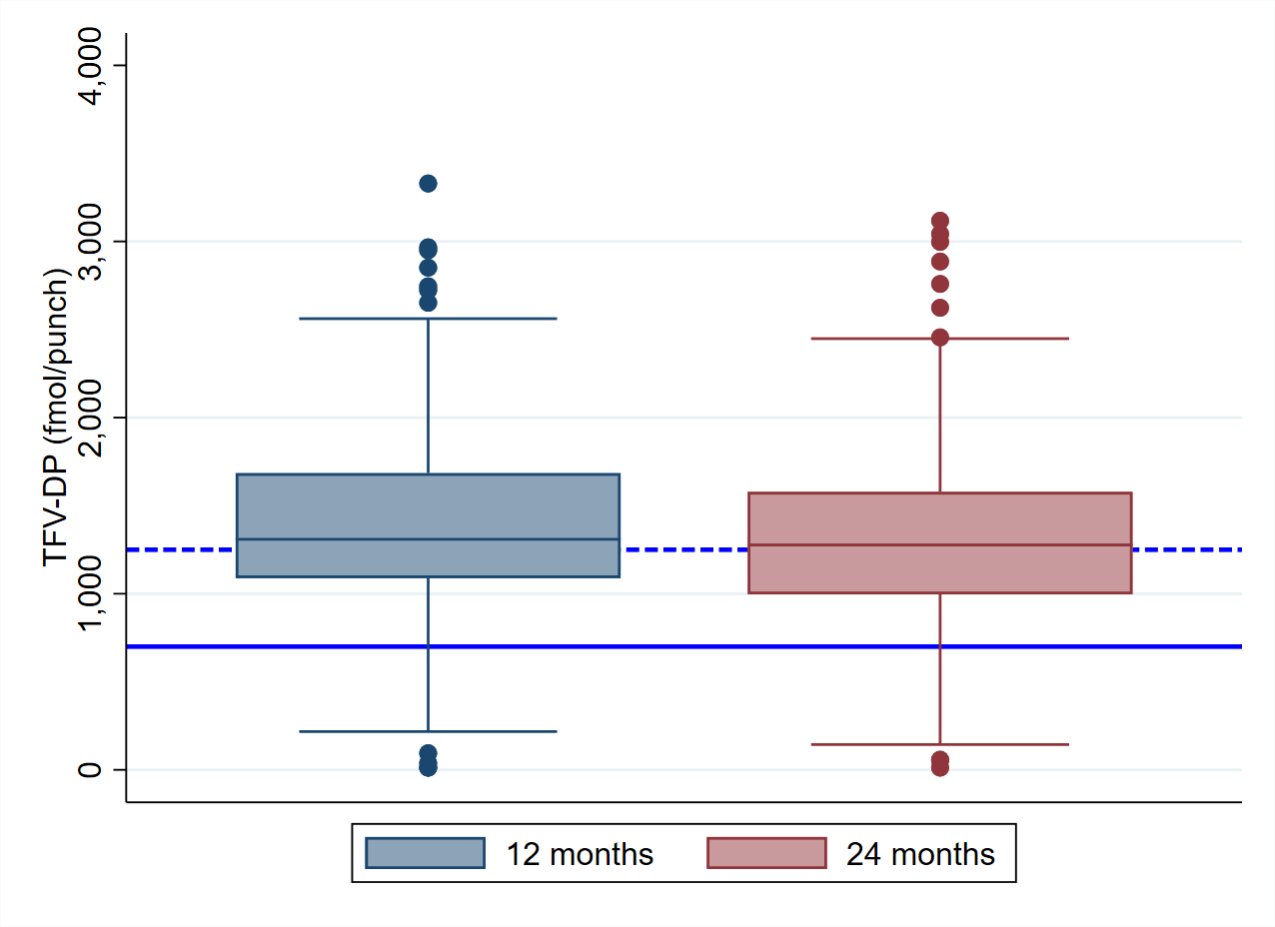
Tenofovir diphosphate (TFV-DP) concentrations from dried blood spots of daily pre-exposure prophylaxis (PrEP) users in fmol/punch by study visit: 12 months and 24 months after PrEP initiation. The solid line indicates 700 fmol/punch, suggested by a previous study as the lower bound of protective drug levels.^5^ The dashed line indicates 1250 fmol/punch, corresponding to consistent adherence to daily PrEP.^4^ Box represents 25th–75th percentiles and median, whiskers upper and lower adjacent values and dots outside values. n (12 months)=246, n (24 months)=215. Amsterdam PrEP demonstration project study, Amsterdam, The Netherlands, 2016–2018.

Median overall TFV-DP concentration was 1299 (IQR 1021–1627) fmol/punch; at 12 months the TFV-DP concentration was 1332 (IQR 1087–1687) fmol/punch and at 24 months 1248 (IQR 929–1590) fmol/punch (figure 1).

At 12 months, 93% (230/246) had TFV-DP ≥700 fmol/punch. Among participants under 50 years, this was 92% (175/190) and among those 50 years or older 98% (55/56). At 24 months, 89% (192/215) had TFV-DP ≥700 fmol/punch, 88% (136/155) of those under 50 years and 93% (56/60) of those 50 years and older.

### Factors associated with intracellular TFV-DP concentrations

**Figure 2:**
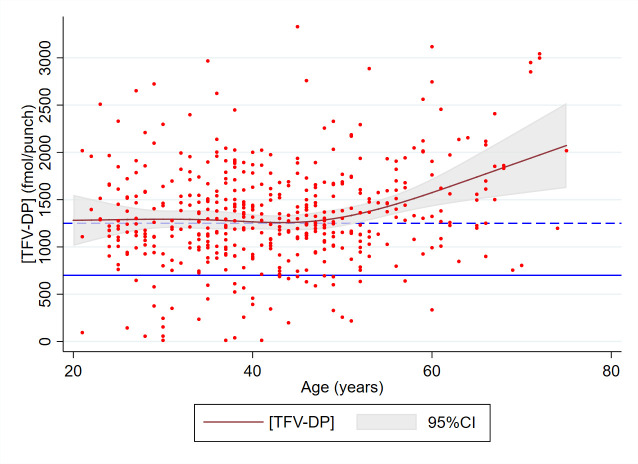
Tenofovir diphosphate (TFV-DP) concentrations from dried blood spots of daily pre-exposure prophylaxis (PrEP) users in fmol/punch (dots) and age modelled as cubic splines (curve). The grey area represents the 95% CI around the modelled estimates. The solid line indicates 700 fmol/punch, suggested by a previous study as the lower bound of protective drug levels.^5^ The dashed line indicates 1250 fmol/punch, corresponding to consistent adherence to daily PrEP.^4^ Amsterdam PrEP demonstration project study, Amsterdam, The Netherlands, 2016–2018.

Eleven variables with p<0.25 in univariable linear regression analyses were included in the initial multivariable model (table 2). In multivariable linear regression analyses, three variables had a significant positive association: age modelled as cubic splines (p=0.0008; figure 2); having had condomless anal sex with a casual partner in the 6 months prior to inclusion in AMPrEP, +166 fmol/punch (95% CI 36.5 to 296) and having access to the app which provided feedback, +146 fmol/punch (95% CI 28.1 to 263). It should be noted that the positive association between TFV-DP concentrations and age mainly pertains to participants aged over 50 years (figure 2).

**Table 2:** Behavioural and psychosocial characteristics and baseline demographic variables of users of daily oral PrEP (emtricitabine/tenofovir disoproxil), associated with TFV-DP concentrations in linear regression analysis using generalised estimating equations (n=257; total number of DBS samples=461)

	Univariable			Multivariable		
	β*	(95% CI)	P value	β*	(95% CI)	P value
Demographic characteristics						
Age (years, modelled as cubic spline; ref.: 21 years)			0.0040			0.0008
35	3.45	(−273 to 280)		13.3	(−271 to 298)	
50	29.3	(−211 to 270)		51.6	(−196 to 299)	
55	139	(−103 to 382)		168	(−80.5 to 417)	
60	288	(9.48 to 567)		325	(44.0 to 606)	
65	457	(104 to 809)		501	(153 to 849)	
Self-declared ethnicity: non-white	−82.2	(−245 to 80.8)	0.32			
Place of residency in the Netherlands: outside Amsterdam	−45.8	(−171 to 79.2)	0.47			
Education level						
Low, middle, other	0		0.57			
High	−40.5	(−181 to 100)				
Employment†						
Employed	0		0.33			
Unemployed	−65.3	(−388 to 257)				
Other (retired, volunteer, disabled, student)	135	(−53.0 to 322)				
Net monthly income in €‡						
≤1700	0		0.97			
1701–2950	16.6	(−139 to 172)				
>2950	18.5	(−149 to 186)				
Living situation						
Alone	0		0.048			
With partner	10.6	(−116 to 137)				
With parents/Flatmates	−190	(−356 to −23.6)				
Steady relationship§	−21.5	(−139 to 96.4)	0.72			
Sexual preference: not exclusively homosexual¶	155	(6.71 to 302)	0.041			
Sexual behaviour (past 3 months)						
Any STI**	−10.7	(−113 to 91.4)	0.84			
Total number of sex partners (log transformed)††	51.5	(−0.158 to 103)	0.051			
Total number of condomless anal sex acts with casual partners (log transformed)‡‡	35.7	(−6.15 to 77.6)	0.095			
Condomless anal sex with a casual partner (6 months prior to inclusion in AMPrEP)	175	(33.0 to 317)	0.016	166	(36.5 to 296)	0.012
Mental health characteristics and drug use						
Score ≥24 on sexual compulsivity scale†† (indication of sexual compulsivity)	−62.7	(−189 to 63.3)	0.33			
Chemsex§§,‡‡,	−12.9	(−118 to 92.0)	0.81			
MHI-5 score <60††,***	−67.0	(−201 to 67.1)	0.33			
Score ≥8 on Alcohol Use Disorders Identification Test†††	−85.9	(−214 to 41.7)	0.19			
Score ≥8 on Drug Use Disorder Identification Test‡‡‡	−29.2	(−137 to 78.7)	0.60			
Neutral to high concern about acquiring HIV§§§	104	(−70.3 to 278)	0.24			
Very important to prevent HIV§§§	94.0	(−39.9 to 228)	0.17			
Access to mobile application						
Extended app	138	(15.0 to 261)	0.028	146	(28.1 to 263)	0.015
AMPrEP study visit						
24 months vs 12 months	−83.0	(−146 to −19.6)	0.010	−91.5	(−155 to −28.1)	0.0047

AMPrEP study, Amsterdam, 2015–2018.

*TFV-DP in fmol/punch.

†6 missing.

‡27 missing.

§5 missing.

¶2 missing.

**4 missing.

††14 missing.

‡‡16 missing.

§§Use of γ-hydroxybutyrate, γ-butyrolactone, methamphetamine or mephedrone prior to or during sex in the 3 months prior to inclusion into AMPrEP.

¶¶Indication of an anxiety or depressive mood disorder.

***Indication of an anxiety or depressive mood disorder.

†††Indication of an alcohol use disorder, 15 missing.

‡‡‡Indication of a drug use disorder, 15 missing.

§§§Scale 1–7, dichotomised, at baseline.

AMPrEP, Amsterdam PrEP demonstration project; DBS, dried blood spots; MHI-5, Mental Health Inventory-5; PrEP, pre-exposure prophylaxis; TFV-DP, tenofovir diphosphate .

One variable was associated with lower TFV-DP concentrations: 24 months study visit, compared with the 12 months study visit, −91.5 fmol/punch (95% CI −155 to −28.1; table 2).

After exclusion of participants who were registered as switching to edPrEP on the DBS sample visit, 250/257 participants remained with 440/461 DBS. In sensitivity analyses, the same four variables remained significantly associated with comparable effect sizes and no other associated variables could be selected (online supplemental table).

10.1136/sextrans-2022-055499.supp2 Supplementary data

## Discussion

In this study among MSM using dPrEP, we found that TFV-DP concentrations were high, indicating excellent adherence in general. Higher levels were observed in participants aged over 50 years, in those reporting condomless anal sex with a casual partner 6 months prior to PrEP initiation and in those receiving visualised feedback on PrEP use and sexual behaviour via an app. TFV-DP concentrations decreased slightly from the 12-month to the 24-month study visits, but remained above the threshold for good adherence.

Our study confirms previously observed associations between older age and better adherence.^17–21^ In line with our results, high adherence levels in participants aged over 50 years have also been observed in the Australian PrEP Demonstration Project (PRELUDE).^19^ Also, the PrEP Brazil study found a greater proportion of participants aged ≥35 years with TFV-DP ≥700 fmol/punch compared with those aged <35 years.^18^ However, our study shows that such an association is not necessarily relevant: the large majority of younger PrEP users in our cohort had drug levels above the threshold of protection (figure 2). Also, it should be noted that higher TFV-DP concentrations in those with older age might be explained by reduced renal function and tenofovir clearance, which could theoretically elevate TFV-DP concentrations.^22^

We found that TFV-DP concentrations decreased slightly between the 12-month and 24-month follow-up visits (table 2), although for the large majority drug levels remained above the protective threshold (figure 1). Decreasing adherence over time is a notorious problem described for several chronic treatments including HIV treatment and preventative medicine, such as management of cardiovascular disease.^23^ Also other PrEP cohorts have shown decreasing adherence over time, for example, over 1 year of follow-up in the PrEP Brazil^18^ and the PRELUDE^19^ studies.

In this cohort of highly adherent participants, the observed slight decline in adherence from month 12 to month 24 since PrEP initiation may be of little clinical significance. Nevertheless, considering that a decline with longer duration of PrEP use was reported in other studies as well,^18 19^ it is important to monitor and encourage adherence in PrEP users over the course of their PrEP careers. In addition, further research is needed to determine whether this downward trend stabilises or decreases further over time. And, if the latter is the case, we should assess what characterises these poor adherers so they might be reached for adherence-improving interventions.

We furthermore noted an association between higher drug levels and condomless anal sex in the 6 months before PrEP was started, which was one of the study’s inclusion criteria. It should be noted that 95% of participants reported condomless anal sex prior to PrEP initiation, and that condom use decreased during follow-up.^24^ Time-updated variables such as number of sex partners and condomless anal sex in the 3 months before TFV-DP concentration measurement were not associated with higher drug levels. This association suggests people who did not have condomless anal sex before using PrEP might be less adherent than people who did. In the light of decreasing condom use and habituation to condomless sex, this less adherent population might be less aware of their dependency on PrEP for protection against HIV.

The RCT within AMPrEP evaluating the effect of receiving visualised feedback on PrEP use and sexual behaviour via an app on adherence showed no effect on the predetermined dichotomised end point of good versus poor adherence, using a cut-off at 700 fmol/punch.^7^ In the current analysis, we used the actual TVF-DP concentrations as outcome, rather than the dichotomised categorisation. The linear regression analyses did show an association between higher drug levels and receiving visualised feedback via the app (table 2). This is in accordance with research on HIV treatment adherence interventions,^25^ where an RCT evaluating the effects of an app providing visualised feedback on adherence and current level of immunoprotection, lead to higher levels of adherence and lower HIV viral load in the intervention arm.^26^ Other studies using mobile interventions to promote adherence to PrEP via text message reminders have shown varying results^18 27^; no other study specifically assessed the effects of feedback on sexual behaviour and adherence via an app as compared with a more basic app without the feedback.

Other studies also found associations between higher adherence and private clinic attendance, early good adherence, group sex, sex with HIV-positive partners, stimulant use, length of schooling, black ethnicity and having a steady partner; we could not evaluate or confirm these associations in our analyses.^18 19^ Research by Wu *et al* 28 found negative associations between self-reported adherence during the most recent anal intercourse and anxiety/depression and chemsex,^28^ which we could not confirm in these analyses. Wu *et al* also found an association between suboptimal adherence and with switching from dPrEP to edPrEP use. In this study, we could not formally assess this as we only included participants who used dPrEP prior to DBS collection, in view of our outcome measure TFV-DP concentration. However, earlier our group observed associations in edPrEP users between time since PrEP initiation and lower adherence, as well as higher adherence levels in older participants^29^; this suggests that these effects are not regimen-specific.

Strengths of this study include the objective measure used to determine adherence: intracellular TFV-DP concentrations. Due to the long half-life of intracellular TFV-DP, this method measures gradients of adherence as opposed to plasma drug levels, which due to the short half-life are susceptible to white-coat adherence (ie, improved adherence in the days leading up to a (clinic) visit). Adherence may also be measured by registering self-reported adherence. However, this is influenced by recall and social desirability bias and may therefore also be unreliable. Another strength is the relatively long follow-up period of 24 months since PrEP initiation.

We acknowledge the following limitations in this study. First, due to the chosen outcome measure we could only include dPrEP users. Also, we know switching between dPrEP and edPrEP regimens was common^30^ and TFV-DP concentrations in DBS cannot distinguish appropriate edPrEP use from dPrEP non-compliance. However, sensitivity analyses excluding participants who switched to edPrEP on the DBS sampling visit gave the same results. Second, intracellular TFV-DP concentration reflects average adherence over time, but it does not prove that PrEP was taken when needed: that is, at the time of possible exposure to HIV. Third, AMPrEP participants were early adopters, mostly white, middle-aged and highly educated MSM, so caution should be exercised extrapolating these results to other groups or settings. Last, in order to improve PrEP effectiveness we had aimed to determine predictors of adherence, as determinants of poorer adherence use could be used by clinicians to guide their counselling efforts; unfortunately our analyses did not yield such clear-cut predictors.

In conclusion, AMPrEP participants exhibited excellent adherence to dPrEP up to 2 years since PrEP initiation, even though TFV-DP concentrations decreased slightly over time. TFV-DP concentrations were especially high among participants over 50 years of age, those who had condomless anal sex with a casual partner before initiating PrEP and those who had access to an app which provided visualised feedback on PrEP use and sexual behaviour. Clinicians should continue giving attention to PrEP adherence to long-term PrEP users over the course of their PrEP careers. Younger PrEP users might benefit more from counselling, and an app may fulfil a facilitating role improving adherence for this digital generation.

10.1136/sextrans-2022-055499.supp3 Abstract translation This web only file has been produced by the BMJ Publishing Group from an electronic file supplied by the author(s) and has not been edited for content.

## Data Availability

Data are available on reasonable request. The AMPrEP data are owned by the Public Health Service of Amsterdam. Original data can be requested by submitting a study proposal to the steering committee of AMPrEP. The proposal format can be obtained from amprep@ggd.amsterdam.nl. Request for further information can also be submitted through the same email address. The AMPrEP steering committee verifies each proposal for compatibility with general objectives, ethical approval and informed consent forms of the AMPrEP study and potential overlap with ongoing studies. There are no restrictions to obtaining the data and all data requests will be processed in a similar way.
