# Supplementary material for: Determinants of adherence to daily PrEP measured as intracellular tenofovir diphosphate concentrations over 24 months of follow-up among men who have sex with men
Source: Sex Transm Infect. 2022 Sep 5;99(5):303–10. doi: 10.1136/sextrans-2022-055499 (PMC10359585; doi:10.1136/sextrans-2022-055499)
Supplement: Supplementary data [file sextrans-2022-055499supp001.pdf]

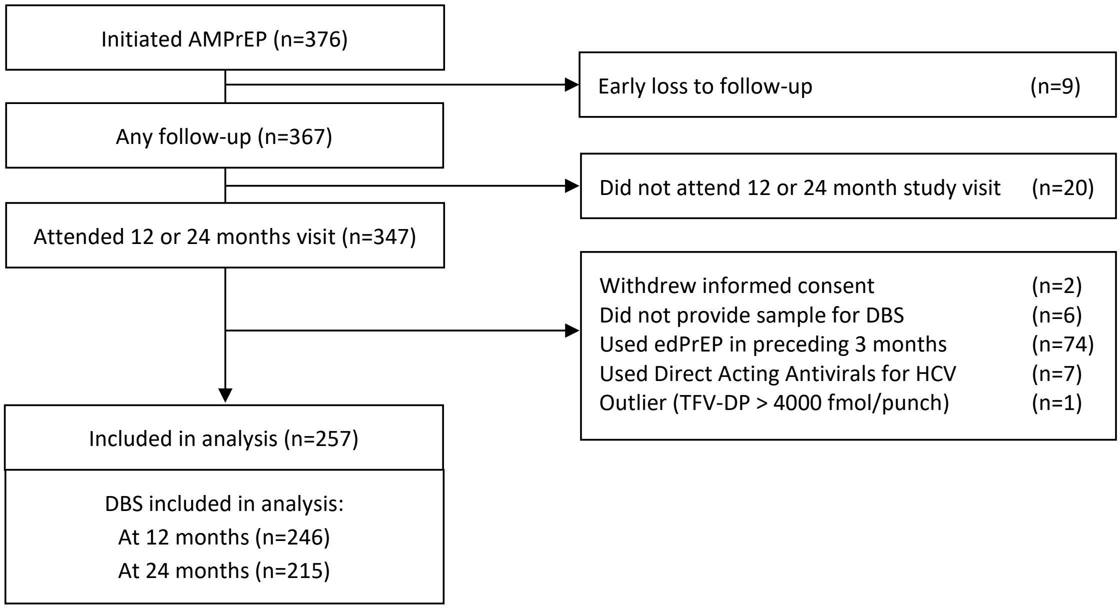

Supplementary Figure: Flow diagram of AMPrEP study participants included in analysis and reasons for exclusion. AMPrEP, Amsterdam, 2015-2018.
AMPrEP: Amsterdam PrEP demonstration project; DBS: dried blood spots; edPrEP: event-driven PrEP; HCV: hepatitis C virus infection; TFV-DP: tenofovir diphosphate concentration in DBS.

20220620 Supplementary Figure Flow Diagram v5
