# Supplementary material for: Determinants of adherence to daily PrEP measured as intracellular tenofovir diphosphate concentrations over 24 months of follow-up among men who have sex with men
Source: Sex Transm Infect. 2022 Sep 5;99(5):303–10. doi: 10.1136/sextrans-2022-055499 (PMC10359585; doi:10.1136/sextrans-2022-055499)
Supplement: Supplementary data [file sextrans-2022-055499supp002.pdf]

Supplementary Table: Behavioural and psychosocial characteristics and baseline demographic variables of users of daily oral PrEP (emtricitabine/tenofovir disoproxil), associated with TFV-DP concentration in linear regression analysis using generalised estimating equations. Sensistivity analysis excluding visits on which participants switched to event-driven PrEP (n=250; total number of DBS samples=440). AMPrEP study, Amsterdam, 2015-2018.

	Univariable			Multivariable		
	β[a]	(95% CI)	p value	β[a]	(95% CI)	p value
**Demographic characteristics**						
Age (modelled as cubic spline; ref.: 21 years)			0.0063			0.0014
35 years	35.4	(-248 to 319)		29.7	(-260 to 319)	
50 years	21.8	(-228 to 271)		28.5	(-226 to 283)	
55 years	131	(-120 to 382)		144	(-110 to 399)	
60 years	288	(1.06 to 576)		308	(21.5 to 595)	
65 years	469	(107 to 832)		496	(141 to 852)	
Self-declared ethnicity: non-white	-88.6	(-253 to 76.0)	0.29			
Place of residency in the Netherlands: outside Amsterdam	-31.4	(-159 to 96.3)	0.63			
Education level						
Low, middle, other	0		0.74			
High	-24.1	(-166 to 118)				
Employment[b]						
Employed	0		0.30			
Unemployed	-123	(-462 to 216)				
Other (retired, volunteer, disabled, student)	129	(-61.8 to 320)				
Net monthly income in Euro's[c]						
≤1700	0		0.97			
1701-2950	44.0	(-117 to 205)				
>2950	39.5	(-132 to 211)				
Living situation						
Alone	0		0.029			
With partner	29.8	(-95.8 to 155)				
With parents/flatmates	-202	(-376 to -28.9)				
Steady relationship[d]	-17.6	(-136 to 101)	0.77			
Sexual preference: not exclusively homosexual[e]	161	(14.5 to 308)	0.031			
**Sexual behaviour (past 3 months)**						
Any sexually transmitted infection[f]	-32.3	(-136 to 71.2)	0.54			
Total number of sex partners (log transformed)[g]	40.1	(-9.92 to 90.1)	0.12			
Total number of condomless anal sex acts with casual partners (log transformed)[h]	25.3	(-16.1 to 66.7)	0.23			
Condomless anal sex with a casual partner (6 months prior to inclusion in AMPrEP)	191	(48.4 to 333)	0.0086	183	(50.5 to 315)	0.0068
**Mental health characteristics and drug use**						
Score ≥24 on Sexual compusivity scale[g] (indication of sexual compulsivity)	-46.9	(-173 to 79.3)	0.47			
Chemsex[h,i]	-5.53	(-105 to 93.6)	0.91			
MHI-5 score <60 [g,j]	-52.3	(-186 to 81.4)	0.44			
Score ≥8 on Alcohol use disorder identification test (AUDIT)[k]	-49.2	(-171 to 73.0)	0.43			
Score ≥8 on Drug use disorder identification test (DUDIT)[l]	-42.7	(-152 to 66.9)	0.58			
Neutral to high concern about acquiring HIV[m]	104	(-70.3 to 278)	0.24			
Very important to prevent HIV[m]	83.2	(-51.9 to 218)	0.23			
**Access to mobile application**						
Extended app	126	(2.01 to 251)	0.046	136	(18.5 to 253)	0.023
**AMPrEP study visit**						
24 months vs. 12 months	-67.7	(-131 to -4.77)	0.035	-76.1	(-139 to -13.4)	0.018

AMPrEP: Amsterdam PrEP demonstration project; DBS: dried blood spots; MHI-5: Mental Health Inventory-5; PrEP: pre-exposure prophylaxis; TFV-DP: tenofovir diphosphate.

[a] TFV-DP concentration in DBS in fmol/punch; [b] 6 missing; [c] 27 missing; [d] 5 missing; [e] 2 missing; [f] 4 missing; [g] 14 missing; [h] 16 missing; [i] Use of γ-hydroxybutyrate, γ-Butyrolactone, methamphatamine or mephedrone prior to or during sex in the 3 months prior to inclusion into AMPrEP; [j] Indication of an anxiety or depressive mood disorder; [k] Indication of an alcohol use disorder, 15 missing; [l] Indication of a drug use disorder, 15 missing; [m] Scale 1-7, dichotomised, at baseline.
