# Supplementary material for: Determinants of adherence to daily PrEP measured as intracellular tenofovir diphosphate concentrations over 24 months of follow-up among men who have sex with men
Source: Sex Transm Infect. 2022 Sep 5;99(5):303–10. doi: 10.1136/sextrans-2022-055499 (PMC10359585; doi:10.1136/sextrans-2022-055499)
Supplement: Abstract translation [file sextrans-2022-055499supp003.pdf]

**Determinanten van therapietrouw aan dagelijkse PrEP bepaald via intracellulaire tenofovir-difosfaat concentraties gedurende 24 maanden follow-up bij mannen die seks hebben met mannen**


**Doelstelling:**

Therapietrouw is cruciaal voor de effectiviteit van orale pre-expositie profylaxe (PrEP) om hiv te voorkomen; daarom wilden we factoren vinden die geassocieerd zijn met therapietrouw bij gebruikers van dagelijkse PrEP (dPrEP).

**Methode:**

Deze studie vond plaats binnen het Amsterdam PrEP demonstratieproject (AMPrEP) bij GGD Amsterdam onder mannen die seks hebben met mannen (MSM). Bij deelnemers die dPrEP (tabletten emtricitabine/tenofovirdisoproxil) gebruikten hebben we bloed afgenomen voor het maken van bloedspotkaarten zowel 12 als 24 maanden na start van PrEP. Uit deze bloedspotkaarten hebben we intracellulaire tenofovirdifosfaat (TFV-DP) concentraties bepaald om de therapietrouw te beoordelen; TFV-DP ≥ 700 fmol/perforatierondje werd als voldoende beschouwd voor bescherming tegen hiv. We hebben associaties van socio-demografische, klinische en gedragskenmerken met TFV-DP-concentraties onderzocht door middel van een multivariabele lineaire regressie.

**Resultaten**

Van de 263 deelnemers die een studiebezoek aflegden op 12 en/of 24 maanden én dPrEP gebruikten, verkregen we 257 (97,7%) bloedspotkaarten van één of beide bezoeken (492 bloedspotkaarten in totaal). De mediane TFV-DP-concentratie was 1299 (interkwartielbereik 1021-1627) fmol/perforatierondje (12 maanden: 1332 [1087-1687]; 24 maanden: 1248 [929-1590]). Hogere TFV-DP-concentraties waren geassocieerd met: hogere leeftijd (p=0,0008), condoomloze anale seks met een losse partner in het halfjaar voorafgaand aan het starten met PrEP (+166 fmol/perforaat; 95% betrouwbaarheidsinterval[CI] 36,5 tot 296) en gebruik van een mobiele applicatie (app) die gevisualiseerde feedback gaf over PrEP-gebruik en seksueel gedrag (+146 fmol/perforaat; 95% CI 28.1 tot 263). Lagere TFV-DP-concentraties waren geassocieerd met een langere duur van PrEP-gebruik (24 vs. 12 maanden; -91.5 fmol/perforaat; 95%CI -155 tot -28.1). Aantal sekspartners, gediagnosticeerde soa en chemsex gedurende de studieperiode waren niet geassocieerd met de TFV-DP-concentratie.

**Conclusie**

Over het algemeen waren de TFV-DP-concentraties hoog bij MSM die dPrEP gebruikten, wat wijst op een uitstekende therapietrouw. Vooral oudere deelnemers, degenen die condoomloze anale seks hadden met een losse partner voorafgaand aan starten met PrEP en degenen die een app met gevisualiseerde feedback gebruikten, vertoonden een betere therapietrouw. Omdat de TFV-DP-concentraties na twee jaar PrEP-gebruik licht waren afgenomen in vergelijking met die na één jaar PrEP-gebruik, benadrukken we het belang van het blijven bespreken van therapietrouw bij PrEP-gebruikers gedurende hun PrEP-carrière.
